# *In vitro *prediction of stop-codon suppression by intravenous gentamicin in patients with cystic fibrosis: a pilot study

**DOI:** 10.1186/1741-7015-5-5

**Published:** 2007-03-29

**Authors:** Isabelle Sermet-Gaudelus, Michel Renouil, Anne Fajac, Laure Bidou, Bastien Parbaille, Sébastien Pierrot, Nolwen Davy, Elise Bismuth, Philippe Reinert, Gérard Lenoir, Jean François Lesure, Jean Pierre Rousset, Aleksander Edelman

**Affiliations:** 1 Centre de Ressources et de Compétence en Mucoviscidose, Hôpital Necker-Enfants Malades, AP-HP, Paris, France; 2 INSERM, U806, Paris, France; 3 Centre de Ressources et de Compétence en Mucoviscidose, Groupe Hospitalier Sud Réunion, Saint Pierre, France; 4 Service d'Histologie-Biologie Tumorale, Hôpital Tenon, AP-HP, UPRES EA 3499, Université Pierre et Marie Curie, Paris, France; 5 IGM, Université Paris-Sud, UMR 8621, Orsay, France; 6 CNRS, Orsay, France; 7 Service d'ORL, Hôpital Necker-Enfants Malades, AP-HP, Paris, France; 8 Centre de Ressources et de Compétence en Mucoviscidose, Centre Hospitalier Intercommunal, 40 Avenue de Verdun, Créteil, France; 9 Centre de Ressources et de Compétence en Mucoviscidose, Hôpital d'Enfants, Saint Denis, France; 10 Université Paris-Descartes, Faculté de Médecine René Descartes, Paris, France

## Abstract

**Background:**

Cystic fibrosis (CF) is caused by mutations in the gene encoding the cystic fibrosis transmembrane conductance regulator (CFTR) protein, which acts as a chloride channel activated by cyclic AMP (cAMP). The most frequent mutation found in 70% of CF patients is F508del, while premature stop mutations are found in about 10% of patients. *In vitro *aminoglycoside antibiotics (e.g. gentamicin) suppress nonsense mutations located in CFTR permitting translation to continue to the natural termination codon. Pharmacologic suppression of stop mutations within the CFTR may be of benefit to a significant number of patients. Our pilot study was conducted to determine whether intravenous gentamicin suppresses stop codons in CF patients and whether it has clinical benefits.

**Methods:**

A dual gene reporter system was used to determine the gentamicin-induced readthrough level of the most frequent stop mutations within the CFTR in the French population. We investigated readthrough efficiency in response to 10 mg/kg once-daily intravenous gentamicin perfusions in patients with and without stop mutations. Respiratory function, sweat chloride concentration, nasal potential difference (NPD) and CFTR expression in nasal epithelial cells were measured at baseline and after 15 days of treatment.

**Results:**

After *in vitro *gentamicin incubation, the readthrough efficiency for the Y122X mutation was at least five times higher than that for G542X, R1162X, and W1282X. In six of the nine patients with the Y122X mutation, CFTR immunodetection showed protein at the membrane of the nasal epithelial cells and the CFTR-dependent Cl^- ^secretion in NPD measurements increased significantly. Respiratory status also improved in these patients, irrespective of the gentamicin sensitivity of the bacteria present in the sputum. Mean sweat chloride concentration decreased significantly and normalised in two patients. Clinical status, NPD and sweat Cl^- ^values did not change in the Y122X patients with no protein expression, in patients with the other stop mutations investigated *in vitro *and those without stop mutations.

**Conclusion:**

Suppression of stop mutations in the CFTR gene with parenteral gentamicin can be predicted *in vitro *and is associated with clinical benefit and significant modification of the CFTR-mediated Cl^- ^transport in nasal and sweat gland epithelium.

## Background

Cystic fibrosis (CF) is caused by mutations in the gene encoding the cystic fibrosis transmembrane conductance regulator (CFTR) protein, which acts as a chloride channel activated by cyclic AMP (cAMP) [1]. More than 1,500 mutations have been described since the discovery of the gene, including premature stop mutations, which are found in about 10% of all CF patients [2]. Pharmacologic suppression of stop mutations within the *CFTR *gene may therefore be of benefit to a significant number of patients.

*In vitro*, aminoglycoside antibiotics suppress nonsense mutations located in the defective *CFTR* by disrupting translational fidelity and thus permit translation to continue to the natural termination codon [3-5]. Moreover, topical application of gentamicin to the nasal epithelium of CF patients with stop mutations restores CFTR function and increases CFTR membrane immunostaining [6]. However, preliminary clinical trials with intravenous gentamicin failed to demonstrate any improvement in patients, suggesting that this molecular effect would not lead to a clinical benefit [7,8]. The variety of mechanisms that modulate readthrough activity may explain this lack of efficacy. Most importantly, the stop codon itself and its surrounding sequences cause variations in the level of gentamicin-induced readthrough [9].

To take these considerations into account, we conducted a two-step study. Using a dual reporter gene assay, we first determined the readthrough level of the most prevalent stop codon mutations in the French CF population after gentamicin incubation. We then focused on the mutations with the best *in vitro *response. To investigate readthrough efficiency after intravenous gentamicin in CF patients with these mutations, in comparison with patients with other stop mutations and without stop mutations, we tested CFTR expression in nasal cells, evaluated the CFTR-mediated chloride secretion in nasal and sweat gland epithelia, and assessed clinical status.

## Methods

### Readthrough quantification in cell culture

A dual gene reporter system was used to quantify the readthrough efficiency directed by the most frequent stop mutations in the French population (Y122X, G542X, R1162X and W1282X) [10], in the presence or absence of gentamicin. It has been previously demonstrated that for a given mutation, the readthrough levels obtained with this experimental system are similar to those obtained *in vivo*[11]. With this dual reporter, all the ribosomes that initiate translation give rise to β-galactosidase activity, while only those that read through the stop codon lead to luciferase activity. The use of a dual reporter allows internal calibration of transfection efficiency, transcription rate of the reporter gene, stability of the message and translation initiation efficacy between independent experiments. A 100% activity control was provided by a construct (TQ) with no stop codon between the coding sequences of the two reporters. Corresponding complementary oligonucleotides were annealed and cloned between the *lacZ *and *luc *coding sequences in the pAC99 vector (Table 1) [11]. NIH3T3 cells were electroporated with 20 μg of amplified plasmid and incubated with or without gentamicin at a concentration of 600 μg/ml. This concentration has previously been shown to be optimal for readthrough induction while being non-toxic during the length of the treatment in a pilot experiment where concentrations ranging from 100 to 1,200 μg/ml where tested on NIH3T3 cells ([11] and Bidou L and Rousset JP, unpublished results). Following transfection, cells were rinsed every day and fresh medium supplemented with antibiotic was added. After three days of expression, cells were harvested and lysed, and β-galactosidase and luciferase enzymatic activities were assayed. The readthrough efficiency was estimated by the ratio of luciferase to β-galactosidase activity obtained with the test construct and normalised with the ratio obtained with the in frame control construct.

### Treatment protocol

The study was approved by the Necker-Enfants Malades Ethics Committee, and written informed consent was obtained for each subject. Exclusion criteria were abnormal baseline hearing or kidney function, nasal polyps, upper respiratory tract infection and treatment by either parenteral or inhaled aminoglycosides in the previous month. The ethics committee refused to authorise inclusion of a control group of patients treated with placebo, holding the opinion that, given the obvious lack of benefit to these patients, the benefits of having a placebo group did not justify their exposure to the risk of parenteral perfusions.

CF patients were treated for 15 days with intravenous gentamicin, administered once daily at 10 mg/kg infused for 30 minutes. Gentamicin trough (24 hours after infusion) and peak (after 30 minutes infusion ended) levels were measured on the third day. The dose was then adjusted to achieve peak serum levels between 20 and 40 μg/ml and trough levels <2 μg/ml. Gentamicin toxicity was monitored before the study and at midpoint by measuring serum creatinine concentrations and by audiometric studies, including pure-tone air conduction threshold and high-frequency tones.

### Treatment endpoints

Clinical evaluation took place the day before gentamicin treatment began (day 0) and the day after it ended (day 15). The Cystic Fibrosis Clinical Score (CFCS), based on pulmonary, nutritional evaluation and temperature [12], was used to assess clinical status. This score has been validated to evaluate modification of clinical parameters. It includes five common symptoms (cough, sputum production, appetite, shortness of breath, and energy level) and five physical findings (temperature, respiration rate, weight, air exchange and crackles). It is graded on a five-point scale for a maximum severity score of 50 points. A decrease of 10 points is considered clinically relevant. Forced expiratory volume (FEV1), forced vital capacity (FVC), and forced expiratory flow at 25 to 75 percent of vital capacity (FEF_25–75_) were expressed as percentages of predicted values for age, sex, and height. Patients had not been treated for 8 hrs with β_2-_mimetics before performing respiratory function explorations. An increase of 7.5% for any of the three tests was considered significant.

Transepithelial nasal potential difference (NPD) was measured according to the procedures described by Knowles and collaborators [13]. Baseline NPD was measured after perfusion of nasal epithelium with saline solution, and NPD changes were recorded after perfusion with the following solutions: 100 μM amiloride in saline solution to block sodium current (Δamiloride), chloride-free solution with 100 μM amiloride (ΔCl^-^free) and chloride-free solution with 100 μM amiloride and 10 μM isoproterenol (Δisoproterenol). The sum of ΔCl^-^free and Δisoproterenol (ΔCl^-^free-isoproterenol) reflects the cAMP activation of nasal mucosa chloride permeability and served as the indicator of transepithelial CFTR-dependent chloride transport.

### CFTR immunocytochemistry

CFTR expression in nasal epithelial cells was assessed with 24-1 (R & D Systems, Lille, France) and MATG 1061 (gift from Transgène, Strasbourg, France) monoclonal antibodies before and after gentamicin treatment. These antibodies recognise amino acids 1377–1480 at the C-terminus and amino acids 503–515 in the first nucleotide-binding domain of CFTR, respectively and were chosen as they are accurate to detect CFTR in nasal epithelial cells by immunocytochemistry [14]. Cells were obtained by nasal brushing below the middle turbinate from the CF patients who agreed to furnish these samples and from two healthy controls thoroughly genotyped for the *CFTR *gene. Cells were spread on slides and fixed at 4°C in acetone for 10 min and then in 4% paraformaldehyde for 20 min. They were then incubated in 10% acetic acid for 10 min. After blockage of nonspecific binding sites with 0.1% TBS, 3% Tween 20, 10% bovine serum albumin-normal human AB serum, cells were incubated for 1 hr with 24-1 or MATG 1061 monoclonal antibodies diluted 1:100 and 1:1,000, respectively. They were then incubated with biotinylated goat-antimouse IgG (Dako, Trappes, France) diluted 1:600 and then with streptavidin-alkaline phosphatase conjugate (Dako, Trappes, France). Enzymatic activity was visualised with Fast Red/naphtol (Sigma Aldrich, St Quentrin Fallavier, France). Slides were then washed, counterstained with hematoxylin and mounted in aqueous medium. Negative controls were obtained by using an isotype-matched mouse nonimmune IgG2 instead of the primary antibody. Two investigators blinded to the patients' data examined all slides. Patients were considered positive for CFTR expression when positive staining at the membrane was observed in at least 10% of the cells examined with at least one antibody.

### Statistical analysis

Data are presented as mean (SD). Data between day 0 (D 0) and day 15 (D 15) were compared with the nonparametric Wilcoxon test. Groups were compared with the nonparametric Mann-Whitney test for quantitative variables. Correlation coefficients were calculated with the Spearman correlation test.

## Results

### Quantification of the readthrough level in cell culture

Basal and gentamicin-induced readthrough levels are shown in Table 1. The R1162X and the G542X mutations yielded basal readthrough of less than 0.03% and increased by a factor of 10 and 15 respectively after gentamicin. The basal readthrough of W1282X was ~10 times higher than those of R1162X and G542X and tripled after gentamicin. Y122X had a basal readthrough level five times higher than that for the W1282X mutation, 22 times that for R1162X and 30 times that for G542X. After gentamicin incubation, Y122X readthrough efficiency remained at least 4.5 times higher than that for W1282X, six times that for G542X and 7.3 that for R1162X. We therefore decided to focus the clinical trial on patients with the Y122X mutation.

### Patients

Table 2 summarises the characteristics of the 18 study patients. Nine carried the Y122X mutation (eight were Y122X homozygous and one was Y122X/F508del) (Group A). Four had another stop mutation: one was homozygous for G542X, one for R1162X, and two were compound heterozygous for W1282X/F508del and R553X/CFTRdele17b (Group B). In addition, five patients without stop mutations were studied according to the same protocol and served as a control group. Three were F508del homozygous, and the other two were compound heterozygous for F508del and class 2 mutations (Group C). There were no significant differences between the three groups for pulmonary status or gentamicin sensitivity of the bacterias in the patient's sputum.

### Clinical response and tolerance

Modification of clinical scores and respiratory function are shown in Tables 3 and 4. Clinical scores for the nine patients with the Y122X mutation (Group A) improved significantly at the end of the study, mainly because of improvements in coughing, sputum production, dyspnea and energy level. Even among the patients with the most severe symptoms, these changes were noted as early as the fourth day of treatment. Pulmonary function also improved significantly for FEV1 and almost at the significant level for FVC and FEF_25–75 _(p = 0.09). These changes were not correlated with microbiological sensitivity to gentamicin. Four of the five patients with significant improvement had bronchial colonisation with gentamicin-resistant bacterias. The most impressive effects were observed in a patient with severe pulmonary disease and bronchial colonisation with a gentamicin-resistant *Burkholderia cepacia *strain. He showed substantial clinical improvement (far greater than with the standard antibiotic treatment) with much less coughing and dyspnea and a considerable fluidification of his sputum. At the end of the trial, he had gained 1.5 kg, and his FEV1 had improved by 19%, FVC by 24%, and FEF_25–75 _by 150% (line 7 in Table 3).

Neither clinical scores nor respiratory functions changed significantly among the patients with other stop mutations (Group B) or among controls (Group C).

Mid-study renal function testing and audiometric studies showed no significant changes from baseline. The mean peak and trough serum gentamicin levels were in the target range for all the patients as early as the third day.

### Sweat test

**Figure 1:**
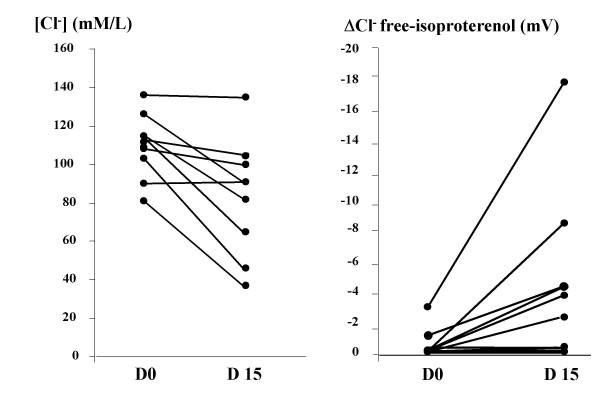
**Sweat chloride concentration and ΔCl^- ^free-isoproterenol before and after gentamicin in Y122X patients (●)**. Previously published representative data of healthy controls (○) are shown [27].

Initial sweat chloride levels were typical for CF patients, that is > 60 mM/L. These levels decreased significantly after treatment among patients with the Y122X mutation (Group A) (Tables 3 and 5; Figure 1). Values normalised in two patients to levels < 60 mM/L, and for another, the value fell from 114 mM/L to 65 mM/L. One week after the study ended in these three patients, sweat chloride concentrations had returned to levels above 75 mM/L, thereby indicating that the gentamicin-induced change did not persist after discontinuation of treatment. There was no significant modification of the sweat chloride values in the two other groups (Table 5).

### Nasal potential difference

**Figure 2:**
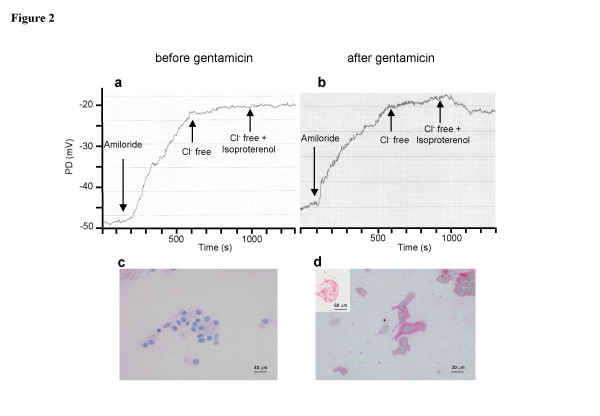
**Example of nasal potential difference tracing (NPD) (a, b) and CFTR immunostaining with MATG 1061 monoclonal antibody of nasal ciliated cells (c,d) before (a,c) and after (b,d) parenteral gentamicin treatment in a Y122X homozygous CF patient**. In (d), insert of nasal epithelial cells from a healthy control subject. Scale bars are 40, 20 and 60 microns for (c), (d) and insert, respectively. (a) NPD before treatment showed a basal NPD of -50 mV, strong depolarisation in response to 100 μM amiloride (25 mV), and no significant response to CFTR activation by 10 μM isoproterenol in chloride-free solution. (b) At the end of treatment, basal PD was close to -45 mV, amiloride-induced depolarisation had not changed, and addition of isoproterenol in chloride-free solution induced hyperpolarisation of ~ -5 mV, indicating a CFTR-dependent chloride transport. (c) No CFTR labeling was observed before treatment. (d) After gentamicin treatment, CFTR labeling was detected in the patient's nasal cells.

NPD values before treatment were typical of CF patients, i.e., highly negative basal NPD, strong depolarisation in response to inhibition of sodium current by 100 μM amiloride (Δamiloride) and no significant response to CFTR activation by isoproterenol 10 μM in chloride-free solution (ΔCl^-^free-isoproterenol) [13] (Figure 2a, Tables 3 and 5). Treatment with gentamicin for 15 days decreased the basal NPD (p = 0.12) and the amplitude of the amiloride-induced depolarisation (p = 0.09), but this did not reach the significance level (Group A, Table 5). Isoproterenol perfusion in chloride-free solution significantly hyperpolarised NPD, from -0.8(1.3) mV to -4.6(6) mV (p = 0.04) (Table 3 and Group A, Table 5). Recordings taken at the end of the study generally showed a stable pre-isoproterenol baseline during low chloride solution perfusion, followed by hyperpolarisation after isoproterenol was added (Figure 2b). There was a significant negative correlation between the changes of Δamiloride and of ΔCl^-^free-isoproterenol. (ρ = -0.9; p = 0.01; Spearman correlation test). These results are consistent with the restoration of a CFTR-dependent secretory chloride transport in the nasal epithelium. Changes in the sweat chloride concentration and response to isoproterenol were not correlated (ρ = 0.17; p = 0.66).

In contrast to the patients with the Y122X mutation, the patients with other stop mutations (Group B) and those without any stop mutations (Group C) had no significant modifications of their basal potential difference or response to amiloride or isoproterenol (Table 5).

### CFTR immunodetection in nasal epithelial cells

The analysis of CFTR expression with the 24-1 and MATG 1061 antibodies were all concordant. The effect of parenteral gentamicin on CFTR was analysed in patients who agreed to nasal brushing, i.e., seven Y122X homozygous patients, one compound F508del/Y122X patient, one R1162X homozygous patient, and the five patients without stop mutations. Before gentamicin treatment, no CFTR labeling was observed in any of the Y122X homozygous patients (see representative picture in Figure 2c), while afterwards, five patients expressed CFTR at the membrane in 10 to 80% of cells (Figure 2d). These five patients were considered responders. The staining pattern did not change for two patients after treatment. Before treatment, cytoplasmic labeling in the compound heterozygous Y122X/ΔF508 patient was found in 20% of the cells with 24-1 antibody and 70% of the cells with MATG 1061 antibody and, after treatment, in 70% and 100% of the cells, respectively. This patient was considered a responder. Among the Y122X responders, CFTR-dependent chloride secretion increased after treatment from -1.1(1) to -5.8(6) (p = 0.06) and remained absent for the two non-responders. Disease also improved among responders. CFCS variation between the two evaluations was -10(7) in the responders versus -2(1) for the non-responders (p = 0.04).

The R1162X patient had no CFTR labeling either before or after treatment (data not shown). The patients without any stop mutation (group C) had only cytoplasmic CFTR staining, a pattern that did not change after treatment (data not shown). Both the R1162X homozygous patient and the group C patients were considered non-responders.

Seric and sputum concentrations were higher in the responder patients (respectively 25.8(2) μg/mL versus 20.6(3.3) μg/mL, p = 0.05; and 2.4(1.4) μg/mL versus 1.7(1.3) μg/mL, NS).

Overall, the pattern of the *in vitro *readthrough, clearly most efficient for the Y122X mutation, was strongly correlated with the immunocytochemically determined CFTR expression in nasal cells, as assessed by the CFTR staining after treatment for the Y122X patients, compared with the lack of staining in both the R1162X patient and the patients without stop mutations.

## Discussion

This open study shows that intravenous delivery of gentamicin at a clinically safe dose can induce readthrough of stop codons. In patients carrying the Y122X mutation, gentamicin treatment resulted in delivery of the CFTR protein at the membrane and in restoration of CFTR-dependent chloride transport in nasal epithelial cells. These changes were correlated with improved respiratory status, thus providing evidence that this pharmacologic therapy may be clinically beneficial. Most interestingly, the clinical, electrophysiological and immunological responses were observed in the patients carrying the mutation that responded best in a reporter gene assay system. This demonstrates that this pharmacologic therapy is mutation-specific and that *in vitro *assays are a useful tool for targeting patients who would benefit from this treatment.

Few studies of CF patients have investigated the potential benefit of gentamicin treatment to suppress premature termination codons, although this mechanism has already been demonstrated *in vitro *[4,5] and in animal models [15]. Wilschanski et al, found that topical administration of gentamicin to the nasal epithelium significantly increased the response of patients with stop mutations to chloride-free isoproterenol solution and the nasal cells CFTR surface staining [6]. Clancy et al tested its systemic effect by treating five CF patients heterozygous for stop mutations with parenteral gentamicin (2.5 mg/kg three times a day) for seven days [7]. The patients with stop mutations had a significantly higher proportion of NPD readings with chloride secretion than the control group with no stop mutations. Sweat tests and lung spirometry did not improve significantly, however. One explanation for the differences between the two studies might be the difference in gentamicin dosage. In the Clancy study, gentamicin dosing was adjusted to achieve peak seric levels from 8 to 10 μg/ml with administration of 2.5 mg/kg three times a day, whereas we aimed peak concentrations from 20 to 40 μg/ml with administration of 10 mg/kg once a day, a dosage regimen known to achieve higher serum concentrations in CF patients without toxic effects. The higher serum concentrations obtained in our study were very likely associated with higher sputum concentrations, because aminoglycoside penetration into bronchial secretions depends on the once daily dose and on the peak serum concentration [16]. This point is important because suppression of stop mutations is dose dependent. This is supported by *in vitro *studies showing that the concentration of full-length CFTR protein after incubation with gentamicin of cells harbouring stop mutations parallels the increase in the antibiotic dose [4]. Studies in *mdx* mice, an animal model for muscular dystrophy, also linked to a stop mutation, further demonstrated that the level of the serum peak was a determinant factor for gentamicin efficacy because mistranslation did not occur below a certain level and was correlated to the antibiotic dose [17]. The fact that seric and sputum concentrations were higher in the responder patients in our study supports the assumption that high concentrations in bronchial secretions might favor the mechanism of readthrough. We therefore hypothesise that a critical concentration is needed to obtain significant readthrough and propose that future clinical studies measure the gentamicin concentration in contact with the target cells to identify the concentration that best promotes useful preferential misreads. We cannot exclude the possibility that the respiratory improvement shown in our patients was due to an antimicrobial effect, even in patients with microorganisms resistant to gentamicin, because of the frequently seen discrepancy between *in vitro *drug sensitivity and in vivo clinical response. However, the fact that patients without any stop mutations did not improve significantly, even those with sensitive strains, provides strong evidence for a clinical effect linked to codon stop suppression rather than to an antibiotic effect. Although there was a correlation in patients carrying the Y122X mutation between the decrease of the response to amiloride (sodium absorption) and the increase of the response to isoproterenol (CFTR dependent chloride secretion), there was only a trend in the decrease of the response to amiloride, the non-significant level probably being due to the small sample of patients. In contrast, patients with mutations producing lower levels of translational readthrough in the cell culture assay (G542X, R1162X and W1282X) did not show significant changes in clinical status, chloride secretion in either the nasal or sweat gland epithelia after gentamicin treatment. Interestingly, the R1162X patient did not have positive protein immunostaining at the end of the treatment, demonstrating a correlation between proteic expression and functional pattern. Systemic administration of gentamicin also significantly modified sweat chloride concentrations, an infrequently seen effect in CF. The absence of correlation between the sweat test, CFTR expression, and function in nasal cells suggests that gentamicin may have different effects on the sweat duct and the respiratory epithelial cell. Parenteral gentamicin may be delivered in lower quantities to sweat glands than to the nasobronchial epithelium. Moreover, sweat tests may not be sensitive enough to detect small changes in CFTR activity.

The originality of our study resides in a multidisciplinary pharmacogenetic approach that allowed us to evaluate the readthrough efficiency first *in vitro *with a dual reporter gene assay and then in CF patients, by measuring clinical, functional, and immunological parameters. Clinical responses for each patient could therefore be interpreted in the light of the level of the gentamicin-induced translational readthrough and the demonstration that the protein synthesised was functional. Thus, as our group is genetically homogeneous, our results are quite convincing, despite the small number of patients.

As stop codons show a broad spectrum of readthrough efficiency in response to gentamicin, we first investigated in culture cells the response to gentamicin of the most frequent stop mutations encountered in French CF patients. The readthrough efficiency for the Y122X mutation, a nonsense mutation mainly found among inhabitants of the Reunion Island and resulting in an ochre termination codon (UAA) [18], was at least five times higher than that for the other CFTR stop mutations tested and more generally, 10 to 40 times higher than that for other previously tested TAA-coded mutations [11]. Several studies have shown that UAA stop codons yield the lowest readthrough levels, with or without gentamicin [19]. The high readthrough levels observed for the Y122X mutation, both basal and induced, suggest that the nucleotide environment of the stop codon may overcome the strong termination efficiency directed by the UAA codon. The +4C nucleotide could account for the better response to gentamicin, because it is associated with greater readthrough efficiency, whereas the other mutations tested in our study, R1162X and G542X, imply a +4G nucleotide, which is associated with a poor readthrough [11]. As the readthrough levels obtained with this experimental system for a given mutation are similar to those obtained *in vivo *[11], these results suggested that patients with the Y122X mutation may benefit from gentamicin treatment. We therefore decided to focus the clinical study on patients homozygous for the Y122X mutation and compare them with a group of patients with the other stop mutations we tested *in vitro*, and a control group of patients with no stop mutations.

Clinical response was interpreted in correlation with CFTR expression and the evaluation of the function of the newly synthesised protein. This point is important as some readthroughs may generate complete but not functional proteins. Cell membrane staining with an antibody that recognises the C terminal region of CFTR demonstrated that gentamicin treatment of CF patients with the Y122X mutation can induce the readthrough of this stop mutation and the synthesis of a full-length CFTR protein delivered at the membrane. This "induced" CFTR protein was functional, as demonstrated by the significant increase in the cAMP-dependent chloride secretion of the responders compared with non-responders. It is likely that a low level of CFTR is sufficient to restore normal airway epithelial function, as suggested by the significant change in nasal CFTR-dependent chloride transport in patients with CFTR expression after gentamicin treatment in as few as 10% of their cells. These results are consistent with previous studies showing that the CFTR gene in normal individuals is expressed at low levels of 1–2 transcripts per cell in the epithelium of the nose, trachea, and bronchi [20] and that a low level of normal CFTR is sufficient to restore normal airway epithelial function in gene transfer experiments [21,22]. The absence of response to gentamicin in two of the Y122X patients enrolled may be due to inefficient uptake or distribution of gentamicin in patients' bronchial secretions, depending on individual pharmacokinetic characteristics, but also to inefficiency at any step during the complex mechanism of stop codon readthrough [23]. One explanation could be a variable efficiency of the nonsense-mediated mRNA decay (NMD) pathway in response to gentamicin treatment. Indeed, a considerable variability in the level of CFTR nonsense transcript was found among the patients carrying the W1282X mutation and receiving a topical administration of nasal gentamicin, with a significant correlation between the amount of CFTR nonsense transcripts and the modification of CFTR function after treatment [24].

## Conclusion

Although our data concern only the Y122X genotype, a rare mutation, they can theoretically be generalised. They demonstrate for the first time that pharmacological suppression of CFTR premature stop mutations can restore adequate levels of functional protein and therefore improve the clinical status of CF patients. Moreover, the correlation between the readthrough levels in cell culture and *in vivo *efficiency in a genetically homogeneous group suggests that clinical trials aimed at suppressing stop mutations should be preceded by *in vitro *studies to target the patients most likely to benefit from this pharmacologic therapy. It provides evidence for the potential for pharmacological therapy in patients with stop mutations. Encouraging results have been recently obtained in CF with amikacin [25] as well as PTC124 [26], a promising new drug that, unlike aminoglycosides, has rare and mild side effects. This less-toxic compound might allow pharmacologic therapy to start with diagnosis. These results hold great promise for the treatment of a subset of patients with CF and other diseases caused by premature stop codons.

## Competing interests

The author(s) declare that they have no competing interests.

## Authors' contributions

ISG conceived and coordinated the study, was the principal investigator, made the nasal potential difference studies and wrote the manuscript. MR, ND, EB, JDB, PR, GL and JFL recruited the patients and were investigators of the study. AF carried out the immunochemistry essay. LB, BP and JPR performed the readthrough studies in cell culture. SP made the nasal brushings. JPR and AE were scientific coordinators of the study. All the authors read and approved the final manuscript.

## Pre-publication history

The pre-publication history for this paper can be accessed here:



## Figures and Tables

**Table 1:** Oligonucleotide sequences used in the dual reporter gene assay, corresponding to the Y122X, G542X, R1162X and W1292X mutations and the TQ in frame control. Readthrough level before and after incubation with 600 μg/ml gentamicin.

		Readthrough level (%)*	
Mutation	Oligonucleotides**	0	600 μg/ml gentamicin

Y122X	w 5' CGCTCTATCGCGTAACTAGGCATAGGC 3'; c 5' GCCTATGCCTAGTTACGCGATAGAGCG 3'	0.52	1.6
W1282X	w 5’ AATATAGTTCTTTGAGAAGGTGGAATC 3’ c 5’ GATTCCACCTTCTCAAAGAACTATATT 3’	0.115	0.35
R1162X	w 5' CGATCTGTGAGCTGAGTCTTTAAGTTC 3'; c 5' GAACTTAAAGACTCAGCTCACAGATCG 3'	0.023	0.22
G542X	w 5' ACTTTGCAACAGTGAAGGAAAGCCTTT 3'; c 5' AAAGGCTTCCTTCACTGTTGCAAAGT 3'	0.017	0.26
TQ: in frame control	w 5' GCAGGAACACAACAGCAATTACAG 3' c 5' CTGTAATTGCTGTTGTGTTCCTGC 3'	100	100

*At least five independent experiments were performed with each construct and showed less than 20% variation.

** w and c refer to the sense and antisense strands respectively.

**Table 2:** Characteristics of the study subjects.

Patients	Group A n = 9	Group B n = 4	Group C n = 5	p Group A *vs*. B	p Group A *vs*. C	p Group B *vs*. C
Age	15.4(4.2)	12(1.8)	16.5(1.7)	NS	NS	NS
CFCS	31(8)	26(2)	24(2)	NS	NS	NS
FEV1 (%)	69(21)	80(12)	74(10)	NS	NS	NS
FVC (%)	70(20)	83(7)	84(22)	NS	NS	NS
FEF_25–75 _(%)	46(30)	67(26)	54(26)	NS	NS	NS

Group A: patients with the Y122X stop mutation. Group B: patients with another stop mutation. Group C: patients without any stop mutation. CFCS refers to the Cystic Fibrosis Clinical Score. FEV1, FVC, FEF_25–75 _refer respectively to forced expiratory volume in one second, forced vital capacity, and forced expiratory flow at 25 to 75 percent of vital capacity and are expressed as percentages of predicted values for age, sex, and height. NS: non-significant. Data are described as mean (SD)

**Table 3:** Characteristics of the subjects with stop codon mutations and variation of clinical and functional parameters after treatment with gentamicin.

Genotype	Sputum colonisation	Age (year)	Δscore	FEV1var	FVCvar	FEF_25–75_var	Sweat Cl^- ^at D0	Sweat Cl^- ^at D15	ΔCl^-^free-iso at D0	ΔCl^-^free-iso at D15	ICC
Y122X+/+	SA	11	-4	24	23	31	126	91	0	0	-
Y122X+/+	PA*	16	-2	-12	-6	-15	79	37	NP	0	-
Y122X+/+	PA*,SA	18	-4	2	-2	-8	109	115	0	NP	+
Y122X+/+	PP*	15	-5	25	19	86	90	91	-0.5	0	+
Y122X+/+	PP*	13	-15	18	8	96	103	46	-1.6	-3.8	+
Y122X+/+	SA	22	-13	3	0	7	108	100	-3.7	-17.6	+
Y122X+/+	BC*	21	-22	18	24	150	136	135	0	-4	+
Y122X+/+	PA*	12	-12	3	-9	NP	119	86	0	-8.2	NP
Y122X+/F508del	SA*	10.5	-3	21	21	45	114	65	-1	-3.3	+

R1162X +/+	SA	14	-2	0.4	0	4	116	131	0	0	-
F508del/W1282X	PA	13	-2	15	14	27	103	100	0	-1.3	NP
G542X +/+	SA	11	-4	21	17	20	113	105	0	0	NP
R553X/CFTRdele17b	PA*	10	0	NP	NP	NP	115	NP	-4	NP	NP

PA: *Pseudomonas aeruginosa*; SA: *Staphylococcus aureus*; PP: *Pseudomonas putida*; BC: *Burkholderia cepacia*; * bacteria resistant to gentamicin. FEV1var refers to the ratio (FEV1_D15_~FEV1_D0_)/FEV1_D0; _FVCvar refers to the ratio (FVC_D15_~FVC_D0_)/FVC_D0_. FEF_25–75_var refers to the ratio (FEF_25–75(D15)_~FEF_25–75(D0)_)/FEF_25–75(D0)_. Δscore refers to value of CFCS_D15_-CFCS_D0_. ΔCl^-^free-iso refers to ΔCl^-^free-isoproterenol. ICC: immunocytochemistry. NP: not performed.

**Table 4:** Modification of clinical score and respiratory function tests after gentamicin treatment.

	Group A n = 9			Group B n = 4			Group C n = 5		
	D0	D15	p	D0	D15	p	D0	D15	p

CFCS	30(8)	21(8)	0.007	25(1)	22 (1)	NS	23.5(2)	23(3)	NS
FEV1(L)	1.82(0.8)	2.07(0.8)	0.04	1.68(0.4)	1.77(0.3)	NS	2(0.3)	2.12(0.4)	NS
FVC(L)	2.2(1)	2.36(0.9)	NS	2.08(0.6)	2.14(0.5)	NS	2.93(0.5)	3(0.4)	NS
FEF_25–75_(L)	1.54(1.1)	1.95(1.06)	NS	1.91(0.9)	1.96(0.8)	NS	1.93(1.8)	1.99(1.8)	NS

Group A: patients with the Y122X stop mutation. Group B: patients with another stop mutation. Group C: patients without any stop mutation. CFCS refers to the Cystic Fibrosis Clinical Score. FEV1, FVC, FEF_25–75 _as defined in Table 2 and are expressed as absolute values. Data are described as mean (SD). Comparison with Wilcoxon test.

**Table 5:** Modification of the sweat test and the NPD results after gentamicin treatment.

	Group A n = 9			Group B n = 4			Group C n = 5		
Patients	D0	D15	p	D0	D15	p	D0	D15	p

Sweat chloride (mM/L)	109(17)	85(31)	0.03	110(7)	112(16)	NS	96(1.5)	105(18)	NS
Basal PD	-56(10)	-49(5)	0.12	-53(11)	-50(8)	NS	-52(8)	-52(7)	NS
ΔAmiloride	20(6)	15(7)	0.09	22(15)	20(9)	NS	19(12)	21(13)	NS
ΔCl^-^free-isoproterenol	-0.8(1.3)	-4.6(6)	0.04	-0.2(0.5)	-0.9(1)	NS	0(0.5)	-0.8(1)	NS

Group A: patients with the Y122X stop mutation. Group B: patients with another stop mutation. Group C: patients without any stop mutation. Data are described as mean (SD). Comparison with Wilcoxon test.
