# Correction to: In vitro prediction of stop-codon suppression by intravenous gentamicin in patients with cystic fibrosis: a pilot study

**DOI:** 10.1186/s12916-018-1138-z

**Published:** 2018-08-25

**Authors:** I. Sermet-Gaudelus, M. Renouil, A. Fajac, L. Bidou, B. Parbaille, S. Pierrot, N. Davy, E. Bismuth, P. Reinert, G. Lenoir, J. F. Lesure, J. P. Rousset, A. Edelman

**Affiliations:** Centre de Ressources et de Compétence en Mucoviscidose, Hôpital Necker-Enfants Malades, AP-HP, Paris, France

## Correction

The original article [1] contains errors in Table 1 affecting some of the presented oligonucleotide sequences and readthrough values in Table 1.

**Table 1:** Oligonucleotide sequences used in the dual reporter gene assay, corresponding to the Y122X, G542X, R1162X and W1292X mutations and the TQ in frame control. Readthrough level before and after incubation with 600 μg/ml gentamicin

Mutation	Oligonucleotide	0	600
Y122X	w 5’ TCTATCGCGATTTAACTAGGCATAGGC 3′ c 5’ GCCTATGCCTAGTTAAATCGCGATAGA 3′	0.02	0.12
W1282X	w 5’ACTTTGCAACAGTGAAGGAAAGCCTTT 3′ c 5’AAAGGCTTTCCTTCACTGTTGCAAAGT 3’	0.115	0. 35
R1162X	w 5’CGATCTGTGAGCTGAGTCTTTAAGTTC 3′ c 5’GAACTTAAAGACTCAGCTCACAGATCG 3′	0.023	0.22
G542X	w 5’AATATAGTTCTTTGAGAAGGTGGAATC 3′ c 5’GATTCCACCTTCTCAAAGAACTATATT 3′	0.017	0.26

The corrected Table 1 displayed below shows the correct presentation of the affected oligonucleotide sequences for mutations Y122X, W128X, and G542X, as well as the correct readthrough values in columns ‘0’ and ‘600’ for the Y122X mutation. These corrections also extend to the corresponding data mentioned in the first paragraph of the Results section and Supplemental Table 1. Whatever the mutation, gentamicin induced readthrough remained very moderate which explains the absence of changes in patients harboring G542X, R1162X and W1282X mutation. This is in contrast to Y122X patients, who unexpectedly demonstrated restoration of adequate levels of functional protein *in vivo* inspite of low readthrough levels observed *in vitro*. One explanation could be a lower efficiency of the nonsense mediated mRNA (NMD) pathway in the cells of these patients, which can not be revealed with the dual reporter system.
